# Assessing Sensor Integrity for Nuclear Waste Monitoring Using Graph Neural Networks

**DOI:** 10.3390/s24051580

**Published:** 2024-02-29

**Authors:** Pierre Hembert, Chady Ghnatios, Julien Cotton, Francisco Chinesta

**Affiliations:** 1 PIMM Laboratory, Arts et Métiers Institute of Technology, Centre National de la Recherche Scientifique (CNRS), 151 Boulevard de l’Hôpital, 75013 Paris, France; chady.ghnatios@ensam.eu (C.G.); francisco.chinesta@ensam.eu (F.C.); 2 Andra, French National Radioactive Waste Management Agency, 92298 Châtenay-Malabry, France; julien.cotton@andra.fr

**Keywords:** graph neural network, classification, sensor state, sensor network, nuclear waste monitoring

## Abstract

A deep geological repository for radioactive waste, such as Andra’s Cigéo project, requires long-term (persistent) monitoring. To achieve this goal, data from a network of sensors are acquired. This network is subject to deterioration over time due to environmental effects (radioactivity, mechanical deterioration of the cell, etc.), and it is paramount to assess each sensor’s integrity and ensure data consistency to enable the precise monitoring of the facilities. Graph neural networks (GNNs) are suitable for detecting faulty sensors in complex networks because they accurately depict physical phenomena that occur in a system and take the sensor network’s local structure into consideration in the predictions. In this work, we leveraged the availability of the experimental data acquired in Andra’s Underground Research Laboratory (URL) to train a graph neural network for the assessment of data integrity. The experiment considered in this work emulated the thermal loading of a high-level waste (HLW) demonstrator cell (i.e., the heating of the containment cell by nuclear waste). Using real experiment data acquired in Andra’s URL in a deep geological layer was one of the novelties of this work. The used model was a GNN that inputted the temperature field from the sensors (at the current and past steps) and returned the state of each individual sensor, i.e., faulty or not. The other novelty of this work lay in the application of the GraphSAGE model which was modified with elements of the Graph Net framework to detect faulty sensors, with up to half of the sensors in the network being faulty at once. This proportion of faulty sensors was explained by the use of distributed sensors (optic fiber) and the environmental effects on the cell. The GNNs trained on the experimental data were ultimately compared against other standard classification methods (thresholding, artificial neural networks, etc.), which demonstrated their effectiveness in the assessment of data integrity.

## 1. Introduction

This section explores the context of the research, ranging from the industrial situation regarding nuclear waste storage to the scientific background regarding graph neural networks.

### 1.1. Andra’s Cigéo Project

**Figure 1:**
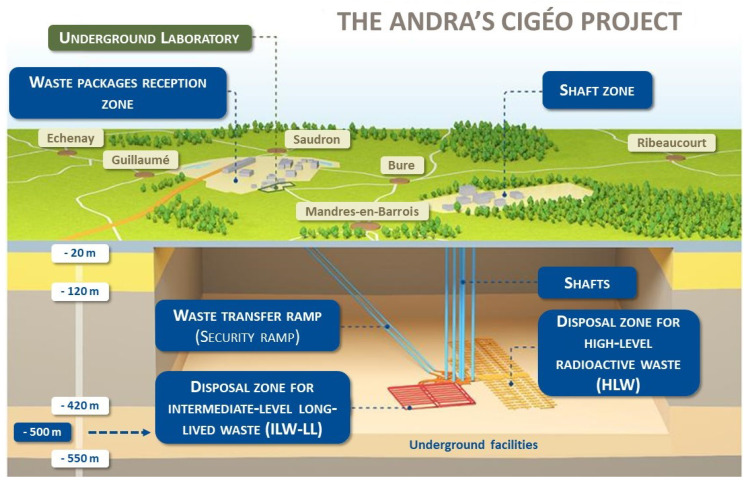
Andra’s Cigéo project.

Radioactive waste storage is a contemporary issue that is addressed in different countries around the globe. One of the most feasible solutions is deep underground storage. However, such storage needs to be actively monitored for safety reasons. This study was part of Andra’s Cigéo project, which aims to design, develop and monitor a deep geological repository for radioactive waste. This repository will store higher-activity waste (HAW), including high-level waste (HLW) and intermediate-level waste (ILW), as shown in Figure 1. Given the dynamic conditions of the storage and the impossibility of accessing the sensors, there is a need for a method that can ensure the consistency of the data. Indeed, the sensor network will evolve over time, in part caused by aging sensors, drifts and failures.

### 1.2. The High-Level Waste (HLW) Demonstrator Cell

**Figure 2:**
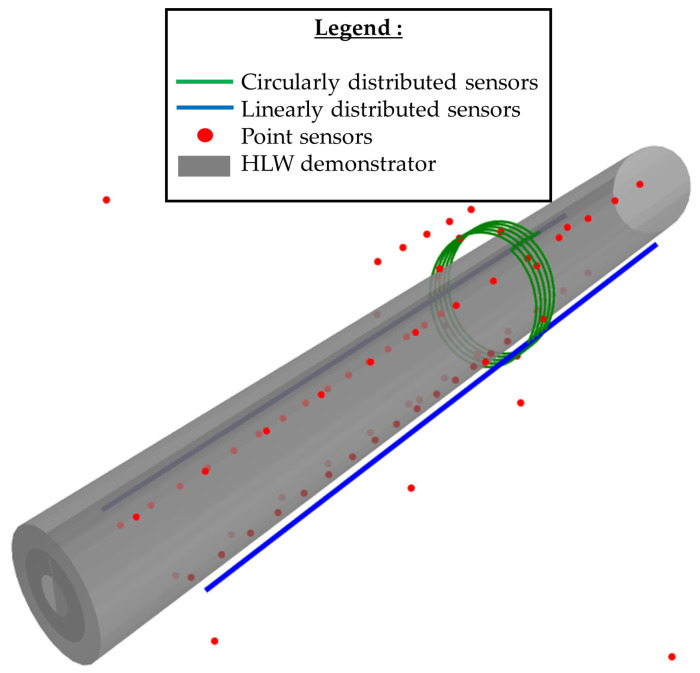
Sensor distribution around the HLW demonstrator cell.

The data used was acquired in Andra’s Underground Research Laboratory (URL), in which there is an HLW demonstrator cell. This demonstrator is a prototype of an HLW storage and is heavily instrumented with both distributed sensors (such as optic fiber) and point sensors. Figure 2 shows the position of each sensor with respect to the demonstrator cell.

**Figure 3:**
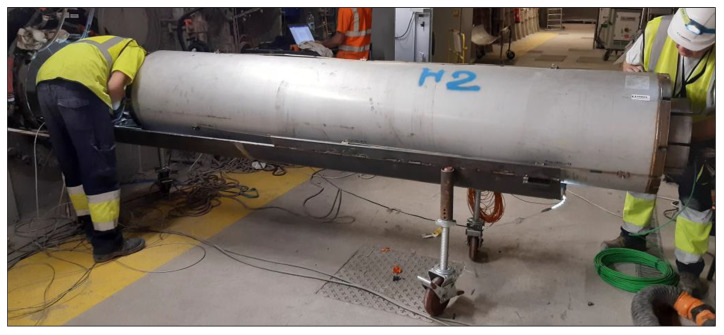
Installation of the thermal source in the HLW demonstrator cell.

This prototype was used in an experiment that consisted of the thermal loading of the cell using the thermal sources presented in Figure 3. This experiment provided us with the data required for the proposed machine learning algorithms. The use of this experimental data was one of the novelties of this work. To the best knowledge of the authors, no other work dealing with sensor integrity assessment around high-level waste demonstrator cells in a deep geological layer has been published in the literature.

### 1.3. Graph Neural Networks and Message Passing

Abbreviations section describes the different notations used in this paper.

The sensor network presented in Figure 2 can be represented as a graph by linking measurement points that are in the vicinity of each other. Moreover, graphs can capture and represent physical phenomena, such as thermal conduction, where energy conservation operates on nodes and heat flows through the graph’s edges. The case studied in this work was one-dimensional, which involved considering only the data of an optic fiber sensor as a unidirectional graph, in order to test the efficiency of a GNN for assessment (classification) of the sensor integrity.

A GNN is a machine learning algorithm that takes a graph as the input and by modifying the graph’s embeddings (from the nodes and the edges), can perform various prediction tasks (i.e., clustering, classification and regression). GNNs can operate predictions at multiple levels [1,2,3,4,5,6,7]:

- At the graph level, for instance, one could give a molecule (as an input graph) and try to find out whether the molecule is toxic.
- At the edge level, typical operations are friend recommendations on a social network graph.
- At the node level, classification tasks can be performed, as was the case in this work, where the state (healthy or faulty) of each sensor (i.e., each node) was derived from the graph of the sensor network.

To perform these predictions, GNNs require the raw data to be updated into more relevant data that are easier to process. Thus, the input graph was updated iteratively using a mechanism called message passing, which was described by Gilmer et al., 2017 [8]. The message-passing mechanism can be divided into the following tasks [1,2,3,4,5,6,7,8,9]:

1. Select a node *v*.
2. Collect information (the messages) from the neighboring nodes N(v) (and edges).
3. Concatenate the messages using a node-order equivariant function α.
4. Update the embedding $x_{v}$ of the node using an update function ϕ (which can be discovered by a neural network). This update function takes the concatenated messages and the selected node’s embedding as the inputs and outputs the updated node’s embedding $x_{v}^{\prime }$.

**Figure 4:**
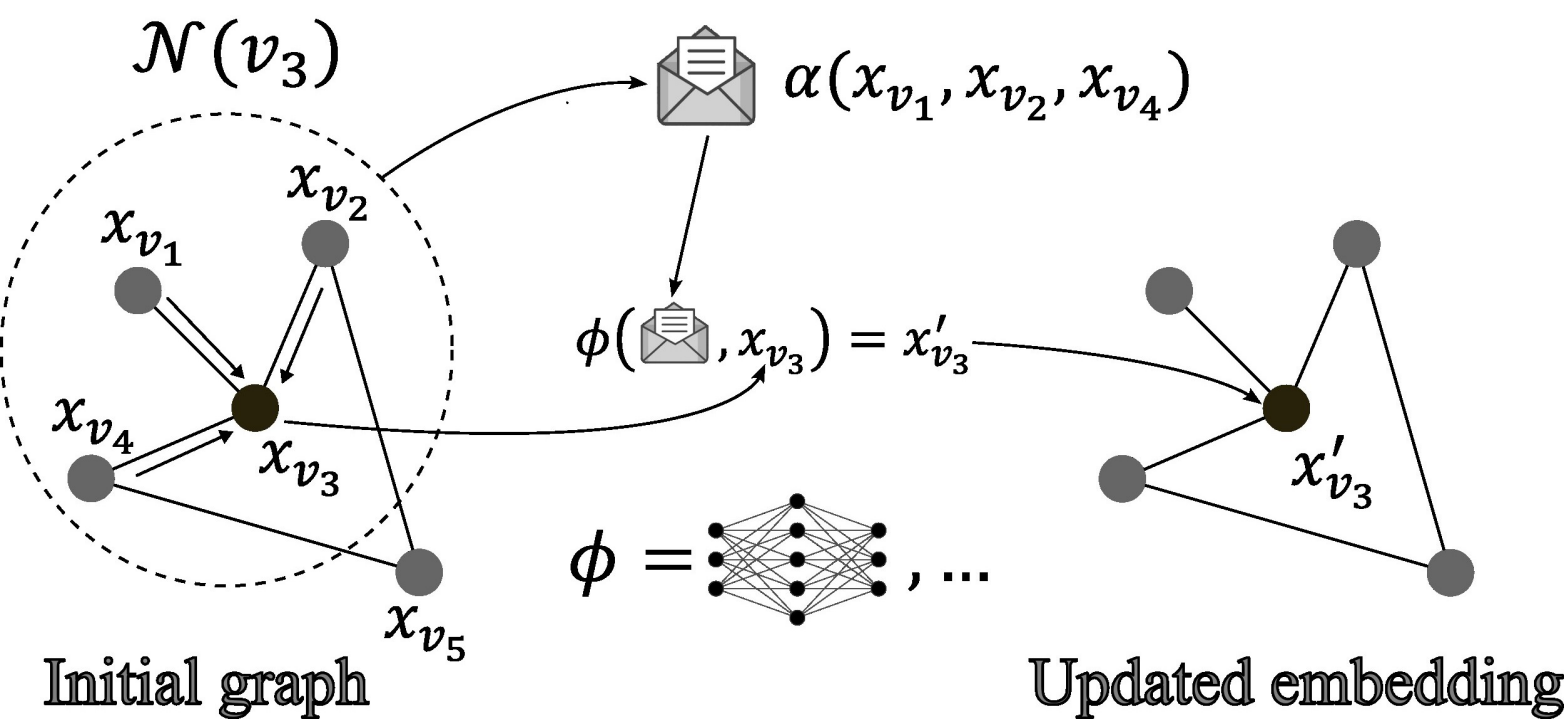
Message-passing mechanism and its use in GNN.

This mechanism, which is presented in Figure 4, leverages the connectivity of the graph by using the information of neighboring nodes (and edges) to update the nodes’ embeddings. Equation (1) describes the updating of a single node’s embedding using the message-passing mechanism [2,8]:

(1) $$x_{v}^{\prime }=\phi \left(\alpha {\left(x_{u}\right)}_{u\in N(v)},x_{v}\right).$$

Note that the update function ϕ and the concatenation function α used in the message-passing algorithm are shared across all nodes [9], which means that the same exact functions are used to update all nodes’ embeddings. This notion is central to the concept of a GNN.

The message-passing mechanism can be understood as a convolution operation on the graph [4,5,6,9,10,11,12,13,14].

A layer of a GNN is created by repeating this mechanism for all the nodes [1,2,4,5,8,9,10]. Indeed, a layer updates all nodes’ embeddings using the message-passing mechanism.

**Figure 5:**
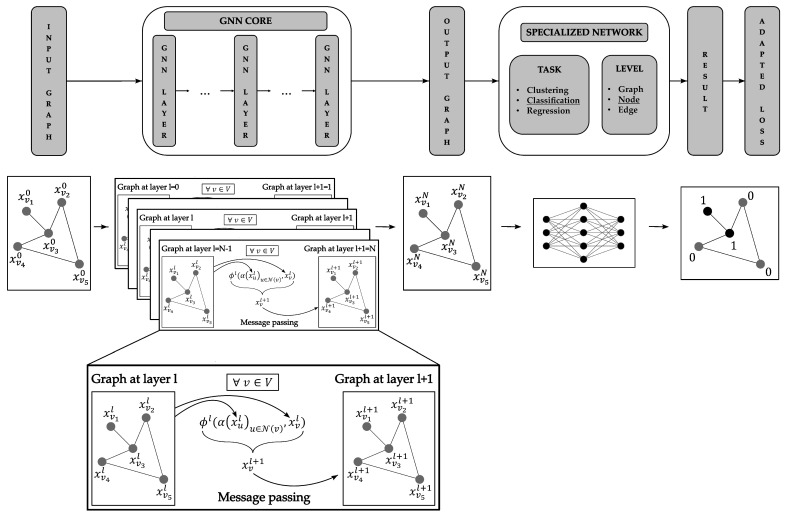
The graph neural network model.

Figure 5 explains how to build a complete GNN: the top part describes the GNN model, and the bottom part showcases its application to a simple graph. This model can be split in two main elements [1,2,4,5,9]:

- The core of a GNN aims at transforming the input graph’s embeddings into embeddings that are easier to interpret by the second part of the model. This is achieved by stacking multiple layers of the GNN, each of which is based on message passing. The connectivity of the graph is not modified during this step. At the bottom of Figure 5, the embeddings $x^{0}$ are transformed into embeddings $x^{n}$ by the repeated application of message passing.
- The specific network takes the updated graph as the input and performs the desired prediction task (in the example, it is node classification). This network’s architecture depends on the related task (pooling layers are used for graph classification, softmax or sigmoid activation functions for classification, etc.). Then, a loss function is applied to the model in order to train it.

Equation (2) is a variation of Equation (1). It describes the updating of the nodes’ embeddings in layer *l* of a GNN. This equation shows the methodology used to obtain the updated embeddings of any node $x_{v}^{l+1}$ (and, by extension, $X_{V}^{l+1}$) given the previous nodes’ embeddings $X_{V}^{l}=\left(x_{v}^{l},v\in V\right)$ [2,8,9]. From now on, this kind of equation is used to describe a GNN.

(2) $$x_{v}^{l+1}=\phi^{l}\left(\alpha {\left(x_{u}^{l}\right)}_{u\in N(v)},x_{v}^{l}\right).$$

### 1.4. Graph Neural Network Models

There exist various models of GNNs [2,3,4,5,6,7,15]. This study focused on a GNN that used convolution operators [2,3,4,5,6,7,15], contrary to the historical recurrent operators [16,17,18,19,20]. Convolution operators can either be spectral [8,9,10,11,12,21,22,23,24,25,26,27,28,29,30] or spatial [13,14,31,32].

A graph convolutional network (GCN) is one of the most straightforward models that use a simple convolution operator presented by Kipf and Welling 2017 [10]. In this model, the update function is the same for all nodes of the graph. Equation (3) presents the GCN model [8,9,10,12,23,24], which uses an activation function σ (often non-linear, such as ReLU) and two matrices $W_{0}^{l}$ and $W_{1}^{l}$ that represent the multi-layer perceptron (MLP) shared across all nodes.

(3) $$x_{v}^{l+1}=\sigma (x_{v}^{l}W_{0}^{l}+\sum_{u\in N(v)}\frac{x_{u}^{l}}{{\left|N\left(u\right)\right|}^{\frac{1}{2}}{\left|N\left(v\right)\right|}^{\frac{1}{2}}}W_{1}^{l}).$$

Equation (2) is the general form of Equation (3).

This model was later expanded by Schlichtkrull et al., 2017 [29], who included different updating functions that depend on the type of connection between two nodes. The latter is called a relational-GCN (R-GCN) because the type of relationship between two nodes induces a different convolution operator. This is prompted by the fact that the relationship connecting two nodes is meaningful and should be taken into account by the GNN. For instance, on Facebook, two users might be connected because they are friends or because they blocked each other, both of which are fundamentally different. Equation (4) presents an R-GCN model using a type of relationship between two nodes noted *r*:

(4) $$x_{v}^{l+1}=\sigma (x_{v}^{l}W_{0}^{l}+\sum_{r\in R}\sum_{u\in N_{r}\left(v\right)}\frac{x_{u}^{l}}{|N_{r}\left(v\right)|}W_{r}^{l}).$$

This model’s main weakness is the increased algorithmic complexity when dealing with a high number of relations. This problem is partially alleviated when using a basis decomposition for $W_{r}^{l}$ or defining $W_{r}^{l}$ with a diagonal block matrix. But even with these improvements, the model is not the most suitable for handling large-sized graphs.

Generalizing even further, graph attention networks (GATs) [31,32,33] use an attention mechanism to operate on the updating function. The strengh of a GAT is that there is no need to have relationship sets; the attention mechanism defines the relationship between two nodes by using their embeddings. Equation (5) presents a GAT model with the attention function $A^{l}\left(x_{u},x_{v}\right)$, which is a learnable parameter:

(5) $$x_{v}^{l+1}=\sigma ([A^{l}\left(x_{v},x_{v}\right)\cdot x_{v}^{l}+\sum_{u\in N(v)}A^{l}\left(x_{u},x_{v}\right)\cdot x_{u}^{l}]W^{l}).$$

Similar to the R-GCN, this model is not easy to apply to large graphs, given its computational complexity.

**Figure 6:**
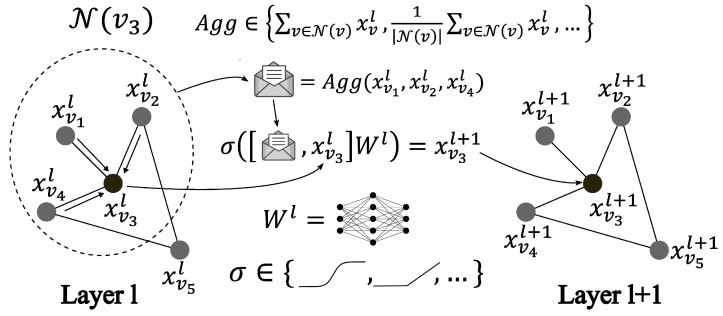
The GraphSAGE model.

The model used in this study for the sensor integrity assessment was a variant of the GCN called GraphSAGE, which was developed by Hamilton et al., 2018 [1,9,13]. This model uses a chosen node order invariant aggregation function (such as a sum, average or max) to collect messages from the neighboring nodes, as shown in Figure 6. This model is efficient when operating on large graphs, which warrants its use.

Equation (6) presents the mathematical model of GraphSAGE, which is similar to Equation (2), with α as the chosen aggregation function (e.g., sum) and $\phi^{l}\left(a,b\right)=\sigma \left(\left[b,a\right]W^{l}\right)$:

(6) $$x_{v}^{l+1}=\sigma ([x_{v}^{l},Agg_{u\in N(v)}\left(x_{u}^{l}\right)]W^{l}).$$

### 1.5. Related Works

The model presented in this work was derived from the GraphSAGE model presented by Hamilton et al., 2018 [13] and aimed at detecting faulty sensors within a network. Therefore, this study built on previous works using GNNs for the following tasks:

- Anomaly detection.
- Node classification.
- Sensor network monitoring.

There are plenty of graph neural networks specialized in anomaly detections [7], whether they are used for IT security [34,35,36,37,38], time series [39] or the industrial use of the Internet of things (IoT) [40].

Node classification is one of the main tasks GNNs can operate [3,4,5,6,7,15,41] and many models have been developed toward this end [42,43,44,45].

Eventually, with sensor-related improvement (IoT, increased precision and compatibility, etc.), sensor network monitoring is being simplified and GNNs have been used for such applications [46,47,48,49,50,51,52,53].

The novelty of the work presented in this paper lay in the combination of these three elements: using a graph neural network for anomaly detection in a sensor network by employing node classification models. Similar work was achieved in Jiang et Luo 2023 [54], where a model for sensor self-diagnosis was used. In Deng et Hoooi 2021 [39], a GNN was developed for the detection of anomalies in time series, which were measured using sensors.

However, both these models use a partial GAT [31], whereas the model proposed in this paper was based on GraphSAGE [13]. The GraphSAGE model has reduced computational costs when dealing with large graphs compared with a GAT, which led to its selection for our application. Another novelty of this work lay in the modification of the GraphSAGE model using elements from the Graph Net framework [2].

## 2. Materials and Methods

This section presents the available data and the modifications applied to generate training datasets for our machine learning algorithms. Then, the used machine learning and comparative classification models are described.

### 2.1. Generating a Training Dataset

**Figure 7:**
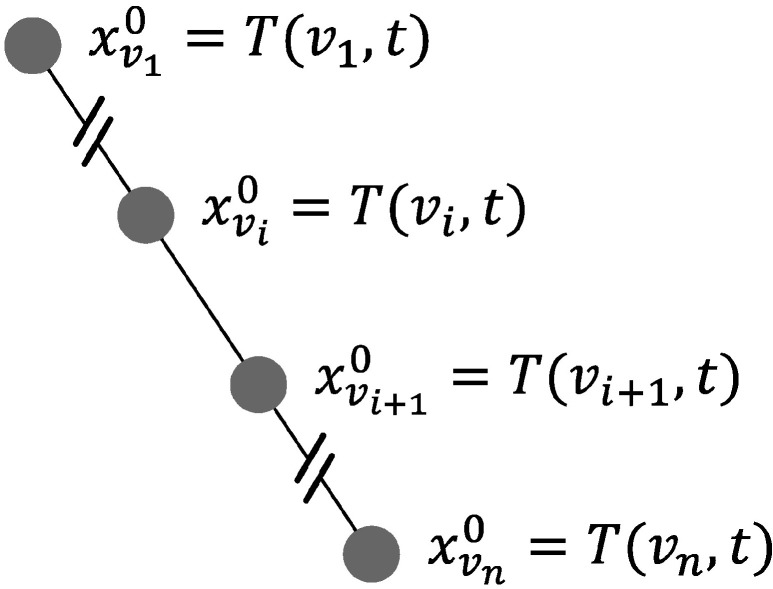
Unidirectional graph representing the data.

As presented in the Introduction, one of the novelties of this study came from the use of industrial data that originated from Andra’s URL. These data represent the responses of a subset of the thermal sensors used in the thermal loading of the HLW demonstrator cell. We collected the responses of one of the distributed optic fiber sensors (Figure 2 in blue), thus inducing a one-dimensional study case. These data could be viewed as a unidirectional graph, as shown in Figure 7.

**Figure 8:**
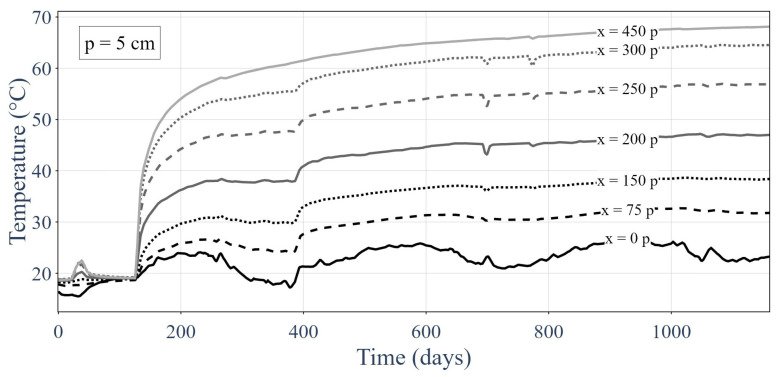
Thermal responses of the optic fiber sensor at set sample points over time.

Figure 8 presents the responses of the distributed sensor over time at various sample points. The lower temperatures were closer to the gallery (i.e., to the cold point) and the higher temperatures were closer to the heat sources.

**Figure 9:**
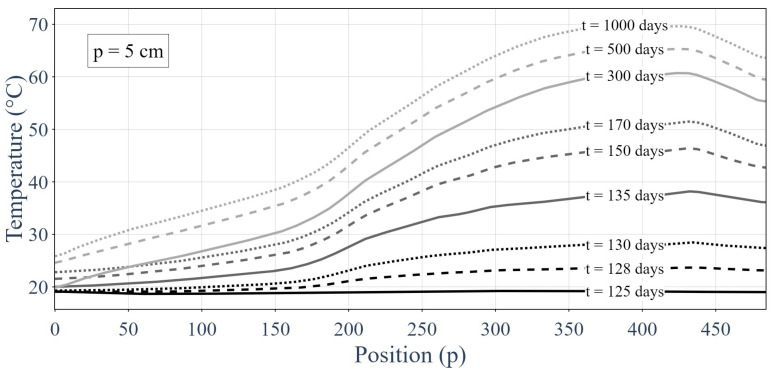
Responses of the integrality of the distributed sensors for different time samples.

Figure 9 shows the temperature along the distributed sensor for different time samples. Similar to Figure 8, we can observe the gallery at x = 0 p (where p is the sensor’s position and consecutive sensors were 5 cm apart), which corresponded to the cold source; heating elements were present in the second half of the HLW demonstrator; and the rock at x = 485 p acted as an insulator (given the small heat flow). The considered time step was one day, while the spatial sampling had a step size of 5 cm.

For the rest of this study, the responses of the distributed optic fiber sensors were considered as a series of point sensors. Thus, distributed sensor failures, such as breaking of the optic fiber, were not modeled, but could be easily inferred. The sensor failures that were considered in this work were partial failures [55,56,57,58,59]:

- Bias.
- Drifting.
- Precision degradation.
- Gain.

The data from the thermal loading of the HLW demonstrator measured by the optic fiber were supposed to have no inaccuracies. Therefore, there was a need to introduce inappropriate responses for some of the sensors to simulate sensor partial failures.

The process used to induce inaccuracies is presented below. Before altering the sensor outputs, a time step was chosen. The sensor responses at the current time step were the starting point of the process that added synthetic errors in the sensors’ outputs.

First, the number of sensors with inaccuracies needed to be determined. This number followed a discrete uniform distribution between 0 and 250 among the 485 measurement points. Equation (7) presents the distribution of the number of sensors that were degraded:

(7) $$n_{degraded}∼U_{D}\left(\left[\;\left[0,250\right]\;\right]\right).$$

Later on, the sensors that were degraded were chosen via an unordered random draw without replacement. This caused one-quarter of the data to be degraded on average, as shown in Equation (8):

(8) $$P\left(v_{faulty}\right)=\sum_{i=0}^{250}\underset{1/251}{\underset{︸}{P(n_{degraded}=i)}}\overset{i/485}{\overset{︷}{P(v_{faulty}|n_{degraded}=i)}}=\frac{250}{990}\approx 25.25\%$$

Once the measurement points to degrade were selected, a mask of sensor integrity was created. This mask returned a Boolean output that described the state of the sensor (i.e., faulty or healthy), as showcased in Equation (9):

(9) $$S_{V}=\left\{S_{v},v\in V\right\}:\forall v\in V,S_{v}=\left\{\begin{array}{c} 0\;\left(\mathrm{healthy}\right)\\ 1\;\left(\mathrm{faulty}\right) \end{array}\right.$$

Inappropriate sensor responses were induced on the previously selected sensors. These inaccuracies were modeled by an offset of a minimum of 2 °C and up to 8 °C, and could be either positive or negative. These offsets followed a continuous uniform distribution, as described in Equation (10):

(10) $$\Delta T∼U_{C}(\left[-8{\;}^{\circ }C,-2{\;}^{\circ }C\right]\cup \left[2{\;}^{\circ }C,8{\;}^{\circ }C\right])$$

**Figure 10:**
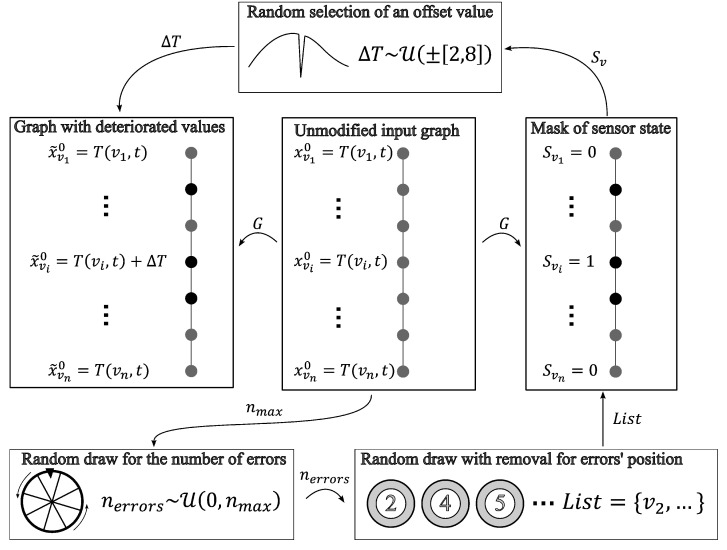
Process of introducing sensor inaccuracies into clean distributed sensor data.

The complete process of inducing inaccuracies in otherwise accurate data is presented in Figure 10.

**Figure 11:**
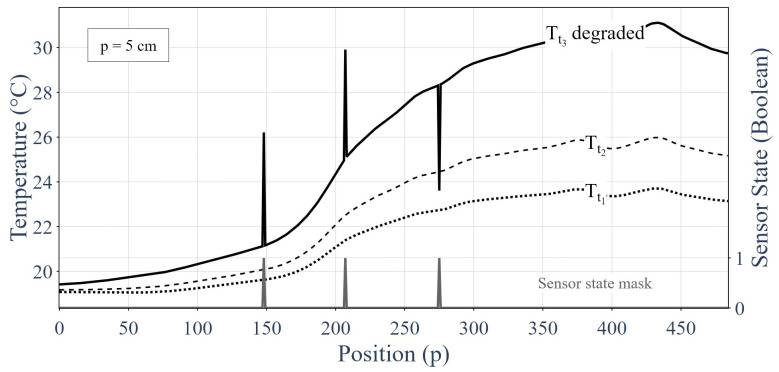
Inputs and outputs of the machine learning algorithm.

Now that the process of adding inaccuracies for simulating sensor degradations has been presented, the inputs and outputs of our machine learning algorithm can be described. Figure 11 represents the inputs (in black) and outputs (in grey) of our model. The input was a graph that contained three successive sensor responses for all measurement points. The responses in the two first time steps (temporally) were unaltered, clean sensor responses, as represented by the two curves with dashes in Figure 11. However, the third graph was based on data that had been degraded by following the aforementioned process and is shown by the full black curve in Figure 10. The expected output was the sensor integrity mask that identified the degraded data, as shown by the grey curve in Figure 11.

The use of three consecutive measurements was based on the integrity of measuring at least two correct values, then incrementally identifying the faulty measures. The two correct initial measurements (the initial condition) gave a baseline to our model.

The measurements at the first time step allowed the model to learn the temperature distribution. The measurements at the second time step enabled the model to learn the evolution of the temperature distribution over time, and therefore, permit a prediction of the temperature distribution at the third time step. Then, the measurements at the third time step introduced sensor degradation.

Using the method presented above, two datasets of a total of 5000 inputs and outputs were created: a training dataset and a testing dataset. These datasets were taken from real measurements performed on the HLW demonstrator shown in Figure 2 and Figure 3. The exact same training and testing datasets were used throughout this study for the different kinds of machine learning algorithms presented. Therefore, a comparison between different machine learning models could be performed on the same testing dataset. Later, we compare the efficiency of different trained models with different combinations of hyperparameters.

### 2.2. The Graph Neural Network Architecture

The GNN used for the node-level assessment of the integrity of the measurements was divided into three successive tasks:

- The creation of the input graph.
- The updating of the graph’s embeddings, i.e., the core of the GNN, using message-passing layers based on the GraphSAGE model.
- The classification of each independent node.

**Figure 12:**
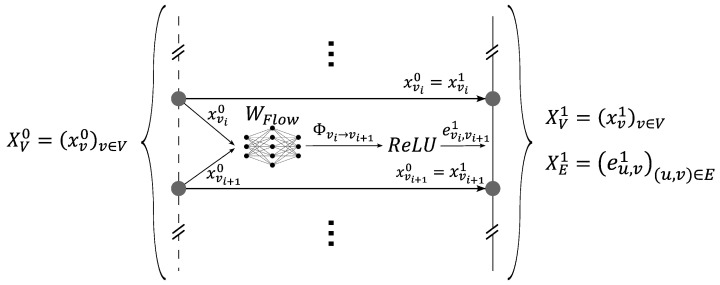
First element of the GNN, which was used to create the input graph.

The first element of the GNN, which is presented in Figure 12, took the unidirectional graph representing the different measurements along the optic fiber considered and added the edges’ embeddings (which could ultimately correspond to the heat flow between each pair of neighboring nodes). The initial graph ($G^{0}$) was composed of three successive time steps, namely, the first two unaltered steps ($T(t_{1})$ and $T(t_{2})$) and the third step with added inaccuracies ($T_{d}\left(t_{3}\right)$). This calculation was performed by a multi-layer perceptron (MLP) $W_{Flow}$ that was applied to neighboring nodes. $W_{Flow}$ was shared across all the edges. The nodes’ embeddings remained unchanged. Equations (11) and (12) model the first part of our GNN. This initialization was aimed at introducing physics by creating edges’ embeddings similar to a physical flow of energy.

(11) $$x_{v}^{1}=x_{v}^{0}=\left(\begin{array}{c} T(v,t_{1})\\ T(v,t_{2})\\ T_{d}\left(v,t_{3}\right) \end{array}\right).$$

(12) $$e_{u,v}^{1}=ReLU\left(\left[x_{u},x_{v}\right]W_{Flow}\right)\;with\;u\in N\left(v\right)\;and\;e_{u,v}^{1}=\left(\begin{array}{c} e_{1}^{1}\left(u,v\right)\\ e_{2}^{1}\left(u,v\right)\\ e_{3}^{1}\left(u,v\right) \end{array}\right).$$

The nodes’ and edges’ embeddings of graphs $G^{0}$ and $G^{1}$ were three-dimensional vectors. Henceforth, all embeddings of any layer *l* of the GNN were three dimensional vectors: $x_{v}^{l}=\left(\begin{array}{c} x_{1}^{l}\left(v\right)\\ x_{2}^{l}\left(v\right)\\ x_{3}^{l}\left(v\right) \end{array}\right)$ and $e_{u,v}^{l}=\left(\begin{array}{c} e_{1}^{l}\left(u,v\right)\\ e_{2}^{l}\left(u,v\right)\\ e_{3}^{l}\left(u,v\right)) \end{array}\right)$.

**Figure 13:**
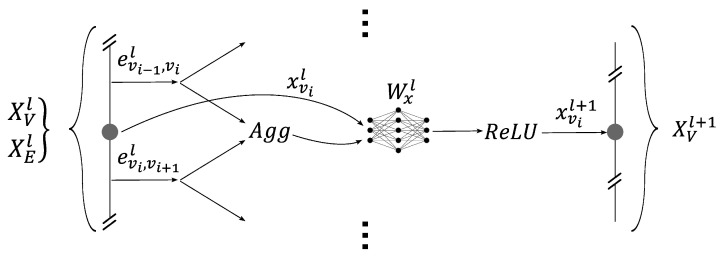
One GNN layer of message passing: updating the nodes’ embeddings.

**Figure 14:**
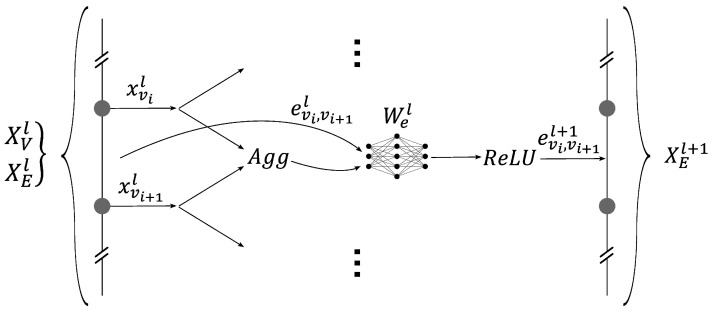
One GNN layer of message passing: updating the edges’ embeddings.

The second element was the core of the GNN. It was used to update the graph embeddings, which made them easier to interpret for the classification network. This element was composed of subsets called GNN layers. Each layer updated the embeddings of the nodes and edges, and by stacking them, the core of the GNN was created. Each layer function used a variation of the GraphSAGE model, as shown by Figure 13 and Figure 14. The novelty of this model lay in the modification of the GraphSAGE model with elements of the Graph Net (GN) framework developed by Battaglia et al., 2018 [2]. This model aims at inferring physics by deducing the flow (on edges) from energy levels (on nodes). And inversely, to apply energy conservation (on nodes) by the cumulation of flows (on edges). The layers update the nodes’ embeddings by aggregating its neighboring edges and vice-versa. Two update functions, namely, $W_{e}^{l}$ and $W_{x}^{l}$, are used to update the edges and the nodes’ embeddings, respectively. These update functions depend on the layer l and are built using a multilayer perceptron (MLP). Moreover they are shared across all nodes and edges. Equations (13) and (14) describe the updating of the nodes and the edges’ embeddings:

(13) $$x_{v}^{l+1}=ReLU([x_{v}^{l},Agg_{u\in N(v)}\left(e_{u,v}^{l}\right)]W_{x}^{l}).$$

(14) $$e_{u,v}^{l+1}=ReLU([e_{u,v}^{l},Agg\left(x_{u}^{l},x_{v}^{l}\right)]W_{e}^{l})\;with\;u\in N\left(v\right).$$

**Figure 15:**
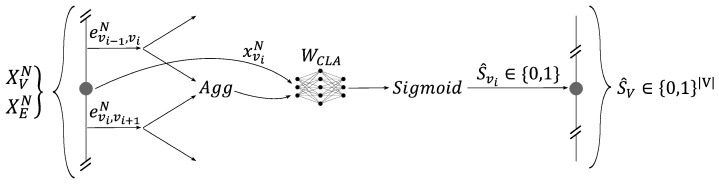
The local classifier predicts the sensor state.

The third element of the network was the classifier presented in Figure 15. Its role was to classify each node with respect to the sensor integrity. This was a local element that, for each node, took the embedding of the corresponding node and the embeddings of its neighboring edges in order to classify the node sensor state as healthy or faulty. This calculation was undertaken by an MLP network denoted as $W_{CLA}$, which was shared across all the nodes. Equation (15) describes the network’s Boolean prediction ${\hat{S}}_{v}$ of the sensor’s state at node v:

(15) $${\hat{S}}_{v}=Sigmoid([x_{v}^{N},Agg_{u\in N(v)}\left(e_{u,v}^{N}\right)]W_{CLA}).$$

The loss function used for this model was a variation of the binary cross-entropy (BCE) [60] described by Equation (16):

(16) $$L\left(S_{V},{\hat{S}}_{V}\right)=\frac{1}{n}\sum_{v\in V}wS_{v}\log\left({\hat{S}}_{v}\right)+\left(1-S_{v}\right)\log\left(1-{\hat{S}}_{v}\right)$$

**Figure 16:**
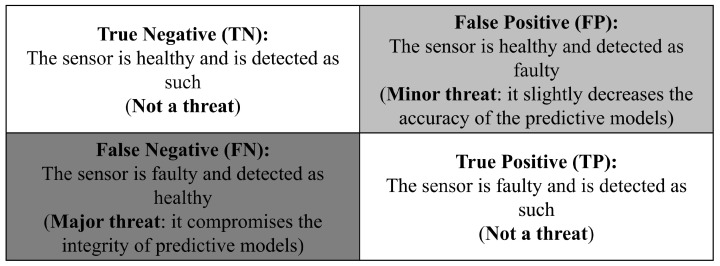
Confusion matrix of our problem.

We used a weight *w* to alter the BCE, with the aim to improve the detection of faulty sensors using our model. Increasing the weight *w* reduced the rate of false negatives (FNs) but increased the rate of false positives (FPs). However, the false negatives (FNs) were the most critical errors in our system, as presented in Figure 16 later in this section.

The different hyperparameters used in defining the GNN were as follows:

- The size of the various MLPs in terms of the number of neurons.
- The aggregation functions used for the message-passing layers and the classifier.
- The number of message-passing layers.
- The weight *w* used in the loss function.

The main idea was to create multiple GNNs with different hyperparameters and to create some kind of benchmark. Table 1 presents all the different hyperparameters tested.

Every combination of the hyperparameters presented in Table 1 was used to train a GNN. In total, there were 162 ($3^{4}\times 2$) unique sets of hyperparameters. For each set of hyperparameters, 10 similar GNNs (for a total of 1620) were trained on the unique training dataset generated following the process presented in Figure 10.

The training parameters were identical for each GNN:

- The used optimizer was the Adam stochastic gradient descent algorithm [61].
- A total of 50 training epochs.
- A batch size of 20 per iteration of the gradient descent algorithm.
- A validation split of 15% (same split for all the networks).

Each GNN was then tested on the unique testing dataset. The results were then displayed in a confusion matrix [62], as presented in Figure 16.

We note that the sensors’ data were used in predictive models later on and if a sensor was detected as faulty, it was not used in the models. As such, false positives (FPs) slightly decreased the efficiency of these models because they lowered the amount of usable data. However, false negatives (FNs) fed wrong information to the predictive models, making them a major threat.

### 2.3. The Thresholding Classification Method

To assess the efficiency of the GNN for the classification of the sensors’ states, a comparison with verified methods, the first of which was the thresholding method described below, was necessary. The used method was based on the same inputs and outputs as the GNN. The method used was decomposed into the following steps:

1. The prediction of the temperature distribution at the third time step without errors was derived from the distributions at the two first time steps. For this purpose, a linear extrapolation method in time was used. Equation (17) presents how to obtain a prediction of the temperature at the third time step.
2. The prediction of the temperature distribution was compared against the deteriorated response (at the third time step) by determining a threshold ε under which the sensor was supposedly healthy and over which the sensor was supposedly faulty. Equation (18) shows how the threshold value was used to assess the sensor integrity.
3. Multiple thresholds ε were tested and the one with the best results was used.

(17) $${\hat{T}}_{3}=T_{2}+\left(T_{2}-T_{1}\right).$$

(18) $$\left\{\begin{array}{c} |{\hat{T}}_{3}-T_{3}|<\varepsilon \Rightarrow S_{v}=0\;\left(healthy\right)\\ |{\hat{T}}_{3}-T_{3}|⩾\varepsilon \Rightarrow S_{v}=1\;\left(faulty\right) \end{array}\right.\cdot$$

### 2.4. The Multi-Layer-Perceptron-Based Classifier

The second classification method used to evaluate the GNN model was a simple feedforward neural network. This neural network inputted the three consecutive temperature responses at one of the graph’s nodes and outputted the sensor state (at said node). Thus, the input and output dimensions were, respectively, three and one.

This multi-layer perceptron was composed of the following dense layers:

1. Layer 1 was composed of 15 neurons and used ReLU as its activation function.
2. Layer 2 was composed of five neurons and used ReLU as its activation function.
3. Layer 3 was composed of one neuron and used sigmoid as its activation function.

This multi-layer perceptron’s training parameters were as follows:

- The optimizer used was the Adam stochastic gradient descent algorithm [61].
- A total of 200 training epochs.
- A batch size of 200 per iteration of the gradient descent algorithm.
- A validation split of 15%.

### 2.5. The Decision Tree Classification Method

The last model used to evaluate the GNN model performance was a decision tree. This model shared the same inputs and outputs as the multi-layer perceptron and the thresholding method. The scikit-learn [63] decision tree model was used.

## 3. Results

This section provides the results from the different methods used on the testing dataset. Different metrics are then given to evaluate the performances of the different methods. Finally, these results are compared with each other.

### 3.1. Results of the GNNs

**Figure 17:**
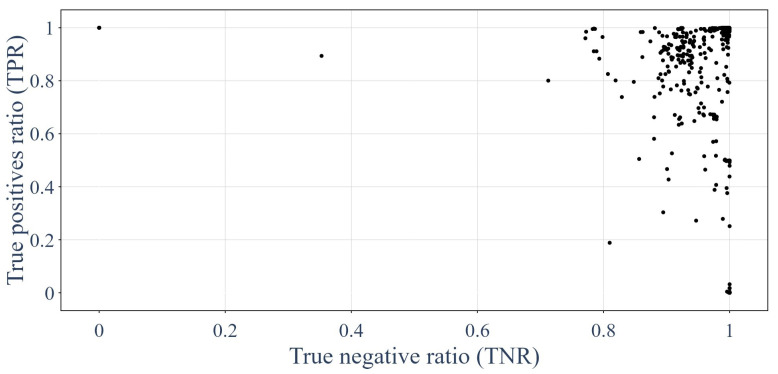
Performances of the GNNs on the testing dataset in terms of recall metrics.

This section shows the results of each of the 1620 GNNs tested on the unique testing dataset. Using the confusion matrix presented in Figure 16, it was possible to use recall metrics [62,64,65,66], such as the true positive rate (TPR) and true negative rate (TNR), to plot the efficiency of each of the GNNs. Figure 17 presents the recall metrics on the testing dataset for each GNN trained. Equations (19) and (20) describe the recall metrics:

(19) $$TPR=\frac{TP}{TP+FN}=\frac{TP}{P}$$

(20) $$TNR=\frac{TN}{TN+FP}=\frac{TN}{N}$$

**Figure 18:**
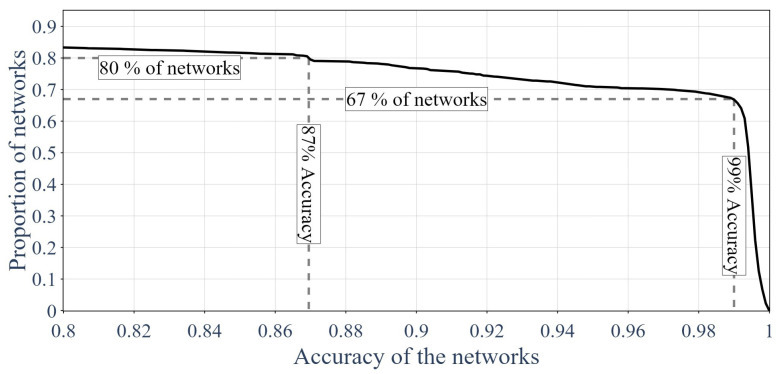
Proportion of GNNs above a certain accuracy.

Then, by using the accuracy metrics [62,64,65,66], it was possible to plot the proportion of GNNs that attained certain accuracy thresholds. This is shown in Figure 18. Equation (21) presents the accuracy metric:

(21) $$Accuracy=\frac{TP+TN}{P+N}=\frac{TP+TN}{n_{sample}}$$

**Figure 19:**
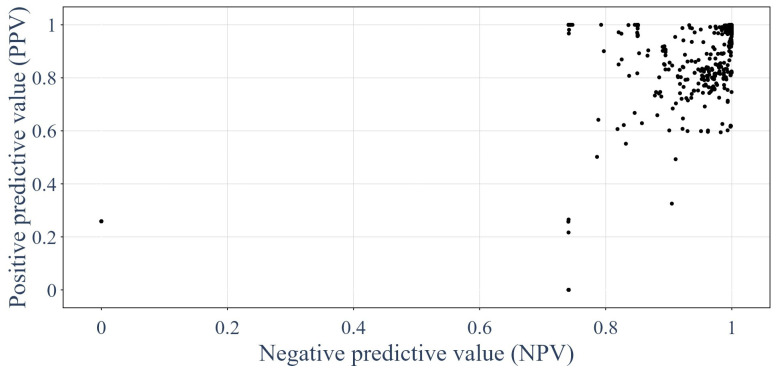
Performances of the GNNs on the testing dataset in terms of precision metrics.

The same analysis could be performed using precision metrics [62,64,65,66], such as positive predictive value (PPV) and negative predictive value (NPV). Figure 19 shows the distribution of the precision metrics on the testing dataset for each GNN tested. Equations (22) and (23) describe the precision metrics:

(22) $$PPV=\frac{TP}{TP+FP}=\frac{TP}{PP}$$

(23) $$NPV=\frac{TN}{TN+FN}=\frac{TN}{PN}$$

**Figure 20:**
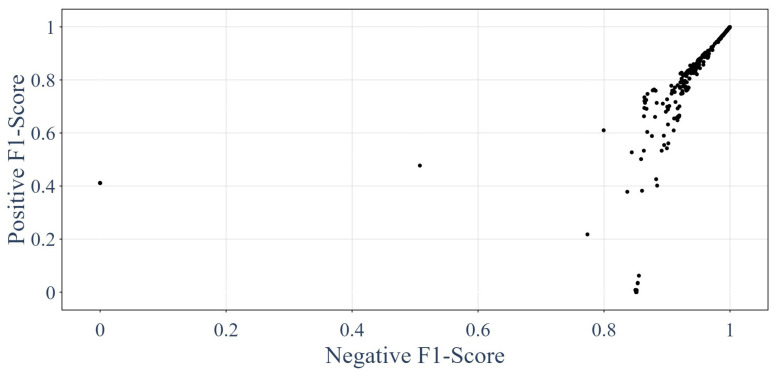
Performances of the GNNs on the testing dataset in terms of F1-scores.

Eventually, the same could be achieved using the F1-scores [62,64,65,66], and the results are presented in Figure 20. The F1-score is the harmonic mean of the recall and precision, as showcased by Equation (24):

(24) $$F1=\frac{2}{recall^{-1}+precision^{-1}}$$

**Figure 21:**
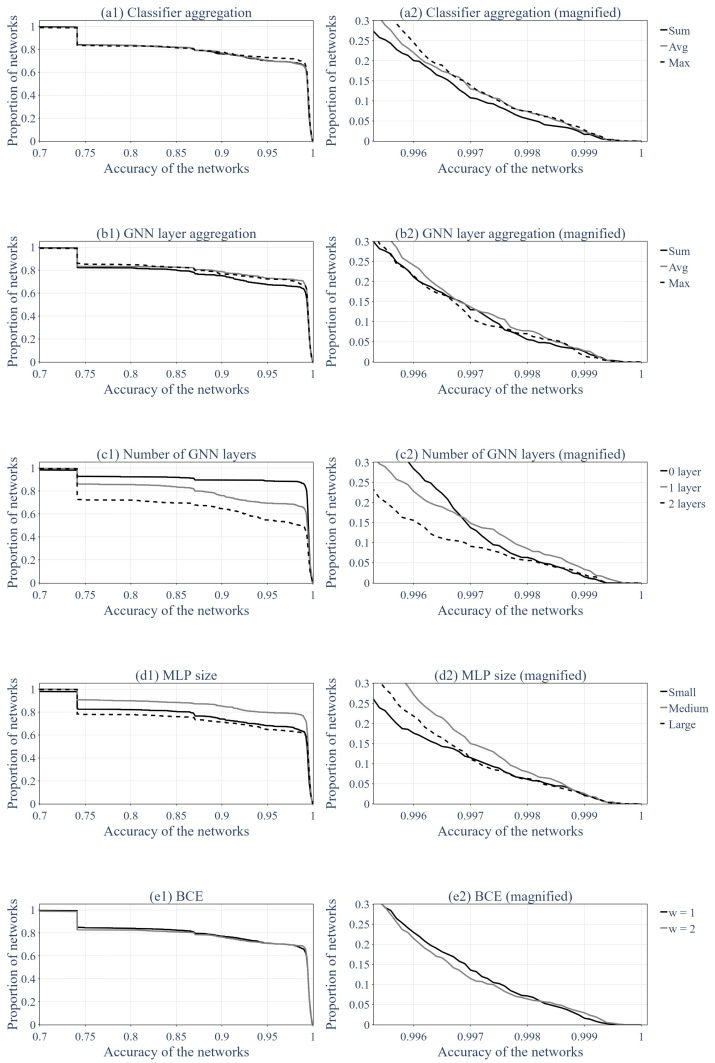
Proportion of GNNs above a certain accuracy, sorted by hyperparameters.

**Figure 22:**
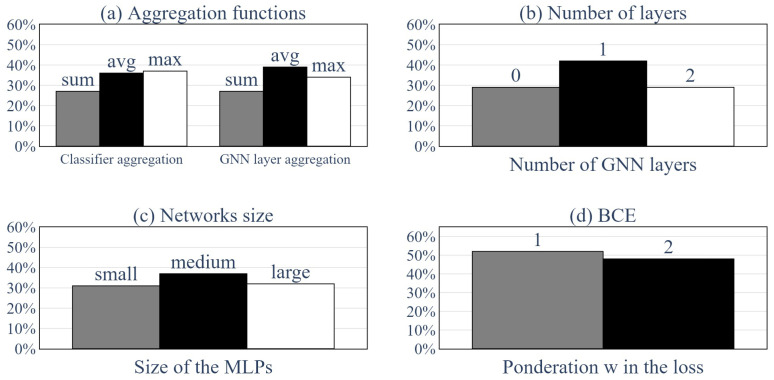
Distribution of the hyperparameters’ set for the 100 top-performing GNNs.

Then, it was interesting to evaluate which hyperparameters presented in the previous section had the most influence on the performances of the GNNs. Figure 21 and Figure 22 present the impacts of the various hyperparameters. Figure 21 shows the proportion of networks that performed over a certain accuracy, similarly to Figure 18, only this time, we split the population into groups with regard to the hyperparameters. Each row of the figure corresponds to a set of hyperparameters (aggregation function, number of layers, etc.), and the subfigure on the right is a magnified view of the figure on the left. This magnified view is meaningful because it showcases which hyperparameter gave the best results when only the best GNNs were taken into account. Thus, the GNNs that did not learn sufficiently were out of the equation.

Figure 22 presents the distribution of hyperparameters for the 100 top-performing GNNs that were trained. Subfigure (a) presents the aggregation functions used for the classification and the message passing, subfigure (b) shows how many layers of message passing the top-performing GNNs used, subfigure (c) shows the sizes of the MLPs used and subfigure (d) presents the *w* coefficient used in the BCE loss function.

### 3.2. Results of the Thresholding

**Figure 23:**
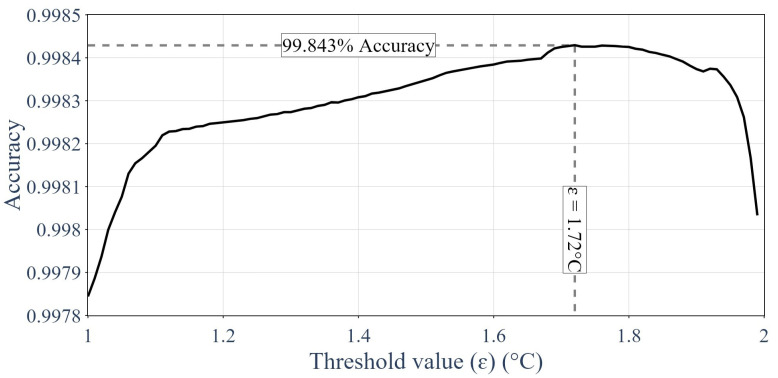
Optimizing the value of ε on the training dataset.

Figure 23 presents the value of ε optimized on the training dataset to provide the best overall performance using the recall metrics.

Later on, the thresholding method was evaluated on the testing dataset using the same threshold ε identified using the training dataset. The confusion matrix of the thresholding method is shown in Table 2.

### 3.3. Results of the Multi-Layer-Perceptron-Based Classifier

Similar to the GNN, the multi-layer-perceptron-based classifier was optimized on the training dataset and was then applied to the test dataset. The confusion matrix associated with this method is shown in Table 3.

### 3.4. Results of the Multi-Layer-Perceptron-Based Classifier

Table 4 presents the confusion matrix that resulted from the application of the decision tree optimized on the training dataset to the test dataset.

### 3.5. Trained GNNs Compared against the Other Classification Methods

Table 5 provides the metrics for the five top-performing GNNs and the other standard classification methods that used the test dataset. The hyperparameters used in the top-performing GNNs are presented in Table 6.

## 4. Discussion

This section explores the results presented in the previous section. It aims to provide an analysis of the hyperparametric study, review the efficiency of the used model and tackle the limitations of the selected GNN architecture.

### 4.1. The Impacts of Different Hyperparameters

The impacts of the different hyperparameters are discussed using Figure 21 and Figure 22. To better understand Figure 21, subfigures 1 on the left and subfigures 2 on the right must be distinguished. Subfigures 1 represent the whole population of trained GNNs for one hyperparameter, whereas subfigures 2 present only the 30% best networks for this hyperparameter. This means that subfigures 1 showcase which hyperparameter had the better odds to produce a GNN with a precision from 70% up to 99%. In contrast, subfigures 2 only focus on networks whose accuracy exceeded 99.5%. The choice between these two options can be made based on the selected objective or precision of the analysis.

First of all, one may choose the hyperparameters that performed the best according to subfigures 1 if the aims are as follows:

- To have a set of hyperparameters that performs well on average.
- To train only a few GNNs and have decent results.

On the other hand, one may choose the hyperparameters that perform the best according to subfigures 2 if the goals are as follows:

- To have a set of hyperparameters that performs the best but many trained GNNs might have relatively bad precision.
- To train a lot of GNNs and pick out the top performers.

Moreover, Figure 22 only presents a distribution of the 100 top-performing GNNs. Thus, it is to be used in the same manner as subfigures 2.

The choice of an aggregation function (both for the classifier and the layers of the GNN) had little impact on the general accuracy of the GNN, as showcased by Figure 21(a1,b1). However, among the top-performing networks, the sum function seemed to perform worse than the max and average functions, as shown in Figure 21(a2,b2) and Figure 22a.

This might have been because the sum was dependent on the size of the neighborhood, whereas the average and maximum were normalized. Indeed, there were two elements in the graph that only had one neighbor (the extremities, i.e., nodes $v_{1}$ and $v_{n}$ of the graph, as shown in Figure 7), while the rest of the nodes had two. This might have been the reason why the networks with sum as its aggregation function underperformed.

Another impactful parameter was the number of layers of message passing used. Figure 21(c1) shows that the networks with zero layers of message passing performed better overall than the networks with one layer of message passing, which, in turn, performed better overall than the networks with two layers of message passing. However, among the top-performing (15% best) networks, the GNNs with one layer of message passing seemed to perform better, as showcased by Figure 21(c2) and Figure 22b.

This result is a bit strange. Indeed, it was expected that the more layers of message passing, the better the result of the GNN. This can be explained by the limited number of epochs because adding another layer of message passing would complexify the optimization of the GNN, and therefore, require more effort and data to learn the correct weights of the network. Moreover, a GNN with a higher number of layers requires optimizing more weights, which increases the risk of falling into a local minimum. Both these phenomena can explain why having fewer layers of message passing increased the overall accuracy but did not transfer to the top-performing GNNs, for which added complexity permitted better results.

Furthermore, Figure 21(d1) suggests that the medium-sized MLPs performed better overall and that the large-sized MLPs performed worse overall. Figure 21(d2) and Figure 22c seem to show the same trend. This could have been the effect of the same phenomena of optimization complexification as exemplified by the number of GNN layers, but this could have also been linked to the inherent dimension of the data. Indeed, using fewer data than the GNN’s inherent dimension limits the ability to learn because the GNN cannot take into account all the problem’s variables, but a higher data dimension can also hinder the ability to learn because the information is too dilute.

Moreover, as expected, when the importance of detecting a faulty sensor was induced by increasing the weight *w* in the BCE formula (Equation (16)), the rate of TPs increased, whereas the rate of TNs decreased.

Finally, this analysis did not take into consideration the correlation between one or more sets of hyperparameters. For instance, small-sized neural networks may perform well with two layers of message passing but not with zero or one. However, this analysis was complex and singling out hyperparameters already showed great empirical results.

### 4.2. Efficiency of GNNs for Sensors’ Integrity Assessment

As presented in Table 5, the most precise GNNs outperformed the thresholding method for every metric, which demonstrated the effectiveness of the model for assessing the sensors’ integrity. However, the GNN had a longer training and a slightly longer prediction time. Although the computation time is a relevent comparison criterion, in this application, the measurements are performed daily, and therefore, there is no need for real-time computation.

When compared with the MLP-based classifier, the most precise GNNs had relatively similar results for all metrics. These results may bring into question the appeal of the use of a GNN for the studied problem; however, it is worth pointing out that the data used here contained very few topological components given they were one-dimensional. Moreover, the temperature profiles of the used experimentation presented in Figure 8 and Figure 9 were rather simple because they involved a relatively uniform heat source. The model for sensor failure was also quite simple (i.e., an offset from 2 °C to 8 °C). The GNN model will outperform by a suitable amount the MLP for a more complex graph, a more complex heat source distributions or a more intricate sensor failure model thanks to its ability to gain insights into the underlying physical information and topology.

The decision tree outperformed the GNN for a majority of the metrics but had a tendency to have an increased amount of false negatives in comparison with the MLP and GNN, which means it might not be the best method for our problem.

In a nutshell, the performance of the method proposed in this work was similar to common classification methods for one-dimensional data that are relatively homogeneous. This guaranteed that the used method was effective for detecting sensor failures.

### 4.3. Upscaling the Model to Complex Networks

The next logical step of this research is to apply this model to more complex sensor networks, for instance, a network composed of all the sensors presented in Figure 2. Two challenges seem to arise from this upscaling: adapting the model to a complex graph and creating the graph of the sensors.

The first can be almost entirely bypassed because the model is based on node-order invariant aggregation functions, such as sum, average or maximum. However, as exposed in the previous subsection, the sum function is not normalized using the neighborhood size, which might cause issues during upscaling. But for the most part, applying the same GNN to a different graph is completely feasible. Moreover, because this model is based on the GraphSAGE model [13], it is really efficient at dealing with large graphs that require fewer computational resources than other GNN models.

The second and main challenge of this upscaling is to create the sensor network’s graph. This task is tricky because we need to define a systematic way to connect various nodes (i.e., sensors) without the graph becoming too dense or too sparse. Indeed, a very dense graph loses topological expressiveness, in addition to being very costly to compute with a GNN. This task is even more complex if we consider that the sensors are not all in the same part of the cell (some are in concrete, others in a cylinder liner, etc.).

### 4.4. Limitations of the Used Model

The first limitation of the used model was linked to the definitions of the sensor failure models, which were considered to be a simple restricted (between 2 °C and 8 °C) offsets in this work. This kind of failure is easier to pinpoint than real errors for two main reasons: first, it is a homogeneous definition, meaning all faults are similar, making them easier to identify; second, it is a simple definition that does not represent all the various ways a sensor can malfunction. This limitation could be lifted by integrating each type of sensor fault separately in the model and by having one class for each type of sensor failure.

The second limitation of the used model was the training complexity, which means that in order to have a GNN with decent predictive capabilities, multiple training sessions are required. It is therefore required to create a list of objective metrics to identify the top-performing networks that operate without biases. This also means that if a new type of fault is identified, the models will need to be retrained in order to be able to measure their performance on this particular problem.

The third limitation was technical and linked to the computational resources required to run a GNN over large graphs, which requires a lot of RAM (random access memory) and processing power. This limitation could be lifted using subgraph learning [67] and by setting up multithreading. Subgraph learning consists of training the GNN on subsets of the initial graph and then reconstructing the network on the whole graph, hence reducing the amount of memory required to store the model.

Another limitation was the use of the GraphSAGE model, which lacks the generalization capabilities of the attention mechanism used in a GAT [31]. However, when dealing with large graphs, the computation of the attention mechanism is very costly. Therefore, there is a tradeoff between the generalization capabilities and the computational resources required for training and predictions.

## 5. Conclusions

In this work, we propose a novel method based on graph neural networks to assess a sensor’s response integrity. The method was applied on real data obtained using Andra’s HLW cell demonstrator. The method was compared to state-of-the-art models (i.e., thresholding, MLP and decision tree) and showed a similar performance. The method could perform even better when dealing with more complex data (from a topological and thermal standpoint). Moreover, the GNN could adapt more easily to various data topologies, which warrants its use for the assessment of sensor integrity for nuclear waste monitoring. Multiple GNNs were trained and compared to find the optimal neural network hyperparameters. The results show that a single message-passing layer was often enough for the selected application, while multiple message-passing layers were harder to train and could result in overfitting.

Future works will deal with the whole sensor network instead of only the data along one of the optic fibers (see Figure 2). The main challenge with this upgrade may be the creation of the sensors’ network, which will have to consider the location of the sensor (i.e., the structural element the sensor is mounted on). In contrast, the scaling of the model from a one-dimensional graph to a complex graph is not a concern since the architecture of the GNN remains similar. Eventually, future works may be directed toward the exploration of multiple GNN models and on the sensor failure models.

Further developments will include interpolation under dynamic conditions (evolution of the sensor network, the medium, etc.) using a GNN. Indeed, once a faulty sensor is identified, it is paramount to evaluate the temperature at this spot to ensure that numerical models can continue to monitor the facility.

## Figures and Tables

**Table 1:** Various hyperparameters used in the benchmark.

Agg MP	Agg CLA	No. of Layers	MLP Size (Flow, Update, CLA) in Number of Neurons	BCE Weight
Sum	Sum	0	Small (2, 15, 15)	1
Avg	Avg	1	Medium (4, 30, 30)	2
Max	Max	2	Large (8, 60, 60)	

**Table 2:** Confusion matrix of the thresholding method that used the test dataset.

TN = 1,791,832	FP = 5240
FN = 555	TP = 627,373

**Table 3:** Confusion matrix of multi-layer-perceptron-based classifier that used the test dataset.

TN = 1,796,452	FP = 620
FN = 133	TP = 627,795

**Table 4:** Confusion matrix of the decision tree classification method that used the test dataset.

TN = 1,796,452	FP = 11
FN = 327	TP = 627,795

**Table 5:** Comparison between the top-performing GNNs and other standard classification methods.

Model	Recall		Precision		F1		Accuracy
	TNR	TPR	NPV	PPV	F1-N	F1-P	
Thresholding	99.708%	99.912%	99.969%	99.172%	99.839%	99.540%	99.761%
MLP	99.965%	99.979%	99.993%	99.901%	99.979%	99.940%	99.969%
Decision tree	99.999%	99.948%	99.982%	99.998%	99.991%	99.973%	99.986%
GNN-1	99.964%	99.975%	99.991%	99.898%	99.978%	99.937%	99.967%
GNN-2	99.975%	99.942%	99.980%	99.927%	99.977%	99.935%	99.966%
GNN-3	99.974%	99.905%	99.967%	99.924%	99.970%	99.914%	99.956%
GNN-4	99.956%	99.950%	99.983%	99.874%	99.969%	99.912%	99.954%
GNN-5	99.954%	99.936%	99.978%	99.869%	99.966%	99.902%	99.949%

**Table 6:** Hyperparameters of the top-performing GNNs.

Model	Agg MP	Agg CLA	N Layers	MLPs’ Size	BCE Weight
GNN-1	Max	Max	1	Medium	2
GNN-2	Avg	Sum	1	Large	1
GNN-3	Sum	Sum	1	Small	2
GNN-4	Sum	Avg	1	Small	2
GNN-5	Max	Avg	1	Large	2

## Data Availability

The data used in this paper are the property of Andra, and therefore, are not available.

## Abbreviations

**Notations**
Variable	Definition
*G*	Graph
*V*	Set of a graph’s nodes
*n*	Number of nodes in the graph (|V|)
*E*	Set of a graph’s edges
*R*	Set of the graph’s relations (edge type)
$u,v,v_{i}$	Individual nodes
(u,v)	Individual edge adjacent to nodes *u* and *v*
*r*	Individual relation
N(v)	Neighborhood of node *v* (subset of *V*)
$N_{r}\left(v\right)$	Neighborhood of node *v* given a relation r (subset of N(v))
*l*	Individual graph neural network layer
*N*	Graph neural network’s final layer
ϕ	General update function
*W*	Weight matrix of a neural network
σ	Non-linear activation function
ReLU	Rectified linear unit function
Sigmoid	Sigmoid function
α	Concatenation function
Agg	Node-order equivariant aggregation function
*A*	Attention function
L	Loss function
$x_{v}^{l}$	Embedding of node *v* at layer *l*
$e_{u,v}^{l}$	Embedding of edge (u,v) at layer *l*
$X_{V}^{l}$	Set of the nodes’ embeddings at layer *l*
$X_{E}^{l}$	Set of the edges’ embeddings at layer *l*
$X_{G}^{l}$	Set of the graph’s embeddings at layer *l*
$S_{v}$	Real sensor state at node *v*
${\hat{S}}_{v}$	Predicted sensor state at node *v*
$S_{V}$	Set of the sensor state’s real value
${\hat{S}}_{V}$	Set of the sensor state’s predicted value
*T*	Temperature in degrees Celsius
$\hat{T}$	Predicted temperature in degrees Celsius
*t*	Time in days
*p*	Sensor’s position
*P*	Probability
$U_{D}$	Discrete uniform distribution
$U_{C}$	Continuous uniform distribution
ε	Threshold value in degrees Celsius
log	Natural logarithm function
**Abbreviations**
GNN	Graph neural network
RecGNN	Recurrent graph neural network
GCN	Graph convolutional network
R-GCN	Relational graph convolutional network
GAT	Graph attention network
MLP	Multi-layer perceptron
URL	Underground Research Laboratory (of Andra)
HLW	High-level radioactive waste
ILW	Intermediate-level radioactive waste
ILW-LL	Intermediate-level long-lived radioactive waste
LLW	Low-level radioactive waste
HAW	High-activity waste
Agg	Aggregation
BCE	Binary cross-entropy
P	Real positive
N	Real negative
PP	Predicted positive
PN	Predicted negative
TN	True negative
TP	True positive
FN	False negative
FP	False positive
TNR	True negative ratio
TPR	True positive ratio
NPV	Negative predictive value
PPV	Positive predictive value
